# Increase in Cd Tolerance through Seed-Borne Endophytic Fungus *Epichloë gansuensis* Affected Root Exudates and Rhizosphere Bacterial Community of *Achnatherum inebrians*

**DOI:** 10.3390/ijms232113094

**Published:** 2022-10-28

**Authors:** Jie Jin, Rong Huang, Jianfeng Wang, Chao Wang, Ronggui Liu, Hanwen Zhang, Maohua Deng, Shicai Li, Xinglu Li, Rong Tang, Chunjie Li

**Affiliations:** 1 State Key Laboratory of Herbage Improvement and Grassland Agro-Ecosystems, Center for Grassland Microbiome, Collaborative Innovation Center for Western Ecological Safety, Key Laboratory of Grassland Livestock Industry Innovation, Ministry of Agriculture and Rural Affairs, Engineering Research Center of Grassland Industry, Ministry of Education, College of Pastoral Agriculture Science and Technology, Lanzhou University, Lanzhou 730000, China; 2 State Key Laboratory of Plateau Ecology and Agriculture, Qinghai University, Xining 810016, China

**Keywords:** endophytic fungi, *Achnatherum inebrians*, bacterial community, root exudates, Cd stress

## Abstract

Soil cadmium (Cd) pollution is a serious environmental problem imperiling food safety and human health. The endophyte *Epichloë gansuensis* can improve the tolerance of *Achnatherum inebrians* to Cd stress. However, it is still unknown whether and how the endophyte helps host plants build up a specific bacterial community when challenged by CdCl_2_. In this study, the responses of the structure and function of bacterial community and root exudates of E+ (*E. gansuensis* infected) and E− (*E. gansuensis* uninfected) plants to Cd stress were investigated. Analysis of bacterial community structure indicated that the rhizosphere bacterial community predominated over the root endosphere bacterial community in enhancing the resistance of CdCl_2_ in a host mediated by *E. gansuensis*. E+ plant strengthened the interspecific cooperation of rhizosphere bacterial species. Moreover, the analysis of root exudates demonstrated *E. gansuensis* and increased the contents of organic acids and amino acids under Cd stress, and most root exudates were significantly correlated with rhizosphere bacteria. These results suggested that *E. gansuensis* employed a specific strategy to recruit distinct rhizosphere bacterial species and relevant functions by affecting root exudates to improve the tolerance of the host to Cd stress. This study provides a firm foundation for the potential application of symbionts in improving phytostabilization efficiency.

## 1. Introduction

Soil cadmium (Cd) pollution is not only the top-ranked inorganic pollutant, but also one of the most toxic pollutants [1]. It inhibits seed germination, disturbs plant photosynthesis and respiration, accelerates reactive oxygen species (ROS) accumulation, and causes irreversible damage to human brains and kidneys through the food chain [2,3]. To protect the ecosystem against its toxic effects, it is necessary to remove soil Cd through a sustainable and effective method. However, most conventional approaches involved in remediation are very expensive and harmful to soil structures [4]. Thus, an environmentally cost-effective technology to remediate Cd-contaminated soil is urgently needed.

Phytostabilization, also called phytorestoration, has been widely accepted as one of the most eco-friendly and low-cost methods to reduce the bioavailability of contaminants in soil [5]. It needs the plant root tissues to uptake and restrain relatively small amounts of heavy metals (HMs) to refrain from extensive erosion, or transform into insoluble substances in the rhizosphere to achieve pollutant immobilization [6]. Based on the above background, forage grasses are regarded as ideal candidates for phytostabilization on account of their HMs resistance, large dry-mass production and extended root system [7]. Several recent studies indicated important potentiality for phytostabilization with various forage grass cultivars in multiple HM (Cu, Cd, Zn and Pb)-contaminated soil [8,9,10]. Although phytoremediation exhibits many benefits over traditional physical and chemical remediation techniques, its application in HM-polluted soils still has several limitations, such as being time-consuming with a slow plant growth rate [11]. A novel phytoremediation strategy concerning plants and their associated microbes is becoming an available coupling method to remove HMs pollution. A wide range of plant-associated microbes, including rhizobacteria and endophytes, have the ability to enhance the host biomass and the efficiency of phytostabilization via different ways, such as providing available nutrients under adverse conditions, producing siderophore to chelate HM, secreting organic acid or phytohormones, etc., [12].

The varied interactions between plants and microbes bring an inevitable question, that is, how do plants recruit their related microorganisms? Kost et al. confirmed that oxalate secreted by lupine and maize was strongly associated with beneficial *Burkholderia* successful colonization [13]. In turn, an *oxc* gene-deletion mutant, which was unable to grow on oxalate, was extensively impaired during the early colonization of both above plants, demonstrating the effects of certain root exudates on the beneficial microbiome. In addition, under certain abiotic stress conditions, some plants change the root exudate composition to increase organic carbon in the rhizosphere, which attracts beneficial microbes [14].

*Achnatherum inebrians* is a native forage grass, and usually grows in harsh environments in Northwest China. Interestingly, *A. inebrians* plants are typical hosts to *Epichloë gansuensis*, a symptomless fungal endophyte, with infections limited to the vertical transmission through seeds of the host plant. Several studies have concentrated much interest on the mechanism of *E. gansuensis* capable of enhancing environment stress tolerance in host plants [15,16,17,18], while others have paid close attention to their powerful ecological function, which alters the structure and diversity of the rhizosphere bacterial community and the root-associated fungal community structure [19,20]. A previous study found that *E. gansuensis* increased the resistance against Cd stress by improving the ability of anti-oxidation in host *A. inebrians* [21]. However, the understanding of whether and how *E. gansuensis* help the host plant build up specific microorganisms in roots when exposed to Cd stress or whether there is certain connection relation between the root exudates and the specific microorganisms to alleviate Cd stress remains limited. Therefore, the aims of our study are to explore: (1) the effects of *E. gansuensis* on the diversity, structure and function of the rhizobacteria and endophyte; (2) the composition of root exudates in *A. inebrians*; and (3) the relationship between root exudates of *A. inebrians* and microbes recruited by *E. gansuensis* in response to Cd stress. This study not only gives a new insight into understanding the endophytic fungi to improve the tolerance of Cd stress in host plants, but also provides a theoretical basis for phytostabilization by using *A. inebrians* and *E. gansuensis* symbionts.

## 2. Results and Discussion

### 2.1. The Effect of E. gansuensis on Biomass and Cd Uptake of Host A. inebrians under CdCl_2_ Stress

**Figure 1:**
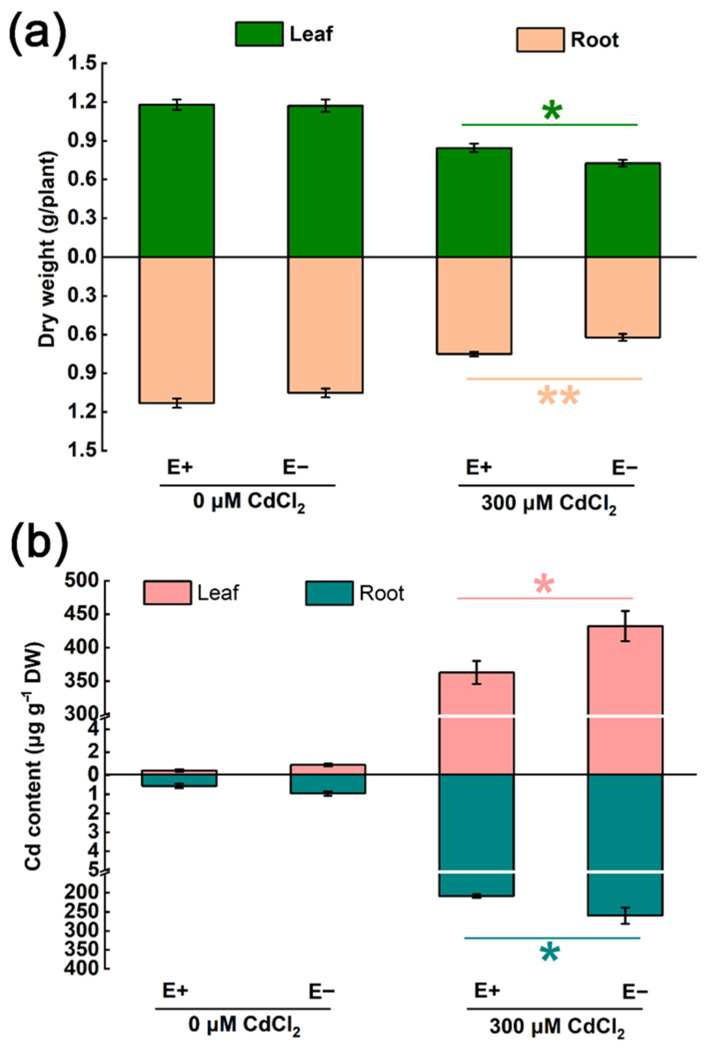
Effect of *E. gansuensis* infection on (**a**) plant dry weight, (**b**) Cd content in plant tissues under 0 and 300 μM CdCl_2_ concentration. Data are mean ± SE (*n* = 5). Asterisks indicated significant difference between E+ and E− plants under different CdCl_2_ conditions (* *p* < 0.05, ** *p* < 0.01).

After the treatment with 300 μM CdCl_2_, both the *A. inebrians* (with and without *E. gansuensis* infection) plants showed significant differences in dry weight and Cd content (Figure 1). Specifically, the dry weight of leaves and roots collected from E+ plants was comparatively higher than those of E− plants under Cd stress, as it increased by 16.3% and 20.9%, respectively (Figure 1a). In contrast, E+ *A. inebrians* had a clearly lower Cd content than E− *A. inebrians,* both in leaves and roots (Figure 1b). No significant variations were observed in plant growth and Cd uptake between E+ and E− plants under the control conditions. The above results indicated that *E. gansuensis* endophyte infection could effectively prevent excessive Cd accumulation, alleviate Cd stress in *A. inebrians* and subsequently promote plant biomass. It has been reported that microbes with powerful assistance in phytoremediation capability can make plants easier to survive in the HM-contaminated environment and improve plant growth [12]. Therefore, the rapid growth rate of the E+ plant and the high adaptability to Cd stress could be conducive to phytostabilization [6].

### 2.2. The Effect of E. gansuensis on the Rhizosphere and Root Endophyte Bacterial Community Structure and Function under CdCl_2_ Stress

#### 2.2.1. Rhizosphere and Root Endophyte Bacterial Community Composition and Diversity

**Figure 2:**
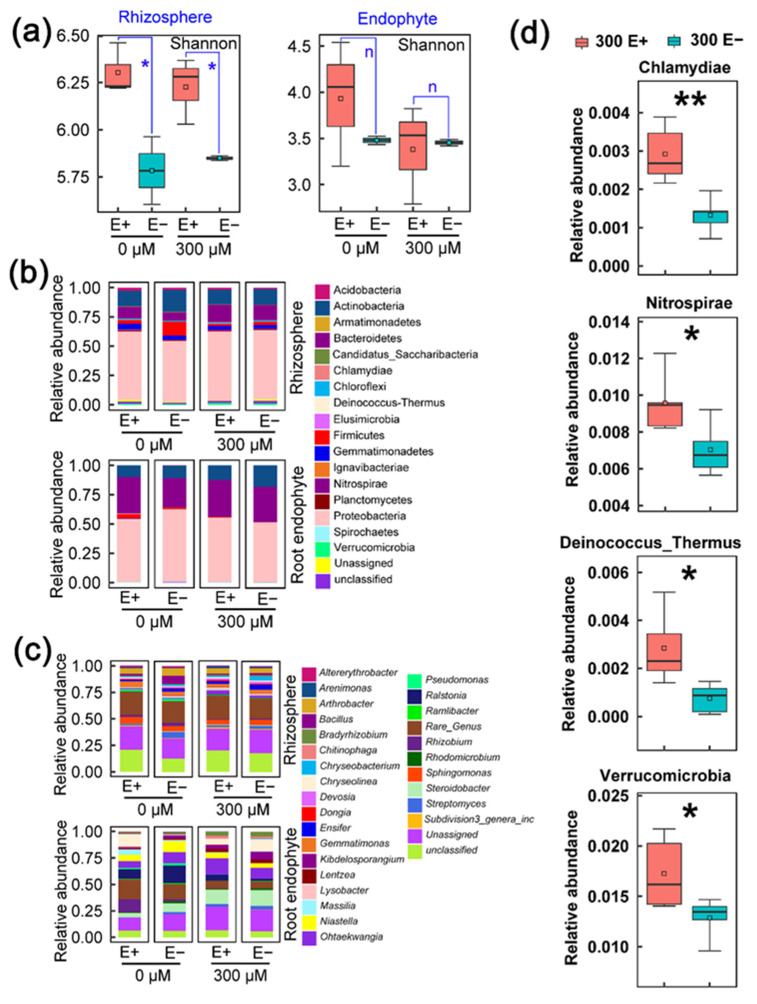
Effect of *E. gansuensis* infection on α-diversity and bacterial community composition of endosphere and rhizosphere under 0 and 300 μM CdCl_2_ concentration. (**a**) Shannon index, (**b**) bacterial community composition at phylum level, (**c**) bacterial community composition at genus level of rhizosphere and root endophytic of E+ and E− plants under different CdCl_2_ conditions. (**d**) Relative abundance of the differential rhizosphere bacterial taxa between E+ and E− plants under 300 μM CdCl_2_ concentration. Asterisks indicated significant difference between E+ and E− plants under different CdCl_2_ conditions (* *p* < 0.05, ** *p* < 0.01).

The microbial community in plant-associated bacteria, including rhizosphere and endophyte, play a vital role in response to HMs stress of plants [22]. To investigate whether the E+ plant induced the variation of rhizosphere and endophyte bacteria, we constructed the 16S rRNA amplicon libraries, followed by Illumina sequencing. The analysis of the α-diversity showed that the Shannon index of rhizosphere bacteria in E+ plant was clearly higher than that of E− plant regardless of 300 μM CdCl_2_ treatment, but there was no significant effect on that of endophyte bacteria (Figure 2a). Similarly, a recent study showed that the accumulation of As and Cd in rice was not correlated with the diversity of the rhizosphere bacterial community [23]. Although bacterial community diversity was not the reason for the observed differences in response to Cd stress between E+ and E− plants, the higher microbial diversity will contribute to the removal of HMs and promote plant growth [12]. This result was consistent with Figure 1 and also reflected the potential phytostabilization capability of E+ plant.

As well as the α-diversity, we further analyzed the bacterial community composition between E+ and E− plants. As shown in Figure 2b,c, the rhizosphere bacterial phyla with higher abundances among all samples were Proteobacteria, Actinobacteria and Bacteroidetes, which accounted for more than 80% of the total sequences. Specifically, the bacterial genera were *Arthrobacter*, *Sphingomonas* and *Gemmatimonas*. For the endophyte bacterial community, it was mainly composed of Proteobacteria, Bacteroidetes and Actinobacteria at the phylum level, which shared over 90% of the total sequences in both E+ and E− plants. *Ohtaekwangia*, *Niastella*, *Steroidobacter* and *Ralstonia* were the dominant genera. We noticed that some bacterial taxa, such as Proteobacteria, Actinobacteria and Bacteroidetes, were consistently enriched in the *A. inebrians* root and rhizosphere. Previous studies have indicated that Bacteroidetes and Actinobacteria can help plants absorb nutrients and promote plant growth under unfavorable environmental conditions [24,25]. Proteobacteria phylum has been described as being highly resistant to HM in metal-contaminated soils [26]. Thus, it implied that Proteobacteria, Actinobacteria and Bacteroidetes play a positive role in *A. inebrians* response to Cd stress. Overall, the relative abundance of abundant phyla both in the endosphere and rhizosphere showed no significant differences between E+ and E− plants under the 300 μM CdCl_2_ treatment.

Difference analysis (*p* < 0.05) of the rhizosphere bacterial phyla showed that the relative abundance of Verrucomicrobia (E+ 1.70% vs. E− 1.20%), Nitrospirae (E+ 0.96% vs. E− 0.70%), Chlamydiae (E+ 0.26% vs. E− 0.10%) and Deinococcus-Thermus (E+ 0.24% vs. E− 0.07%) obviously enhanced in E+ plant under Cd stress compared with E− plants (Figure 2d). Most notably, three-fourths of microbial phyla were rare (relative abundance < 1%) phyla [27]. It was reported that rare species were often associated with multiple functions of the soil ecosystem [28]. Nitrospirae phylum plays a pivotal role in nitrification and improves the availability of soil nutrients, while Deinococcus-Thermus has strong resistance to withstand extremely adverse environments [29,30]. However, the endophyte bacterial rare phyla had no evident difference in all the treatments. These results indicated that rare rhizosphere bacterial species are essential to Cd tolerance of E+ plant. The Nitrospirae, Chlamydiae and Deinococcus-Thermus may potentially be the key taxa of rhizosphere bacteria and play an important role in the phytostabilization of the E+ plant.

**Figure 3:**
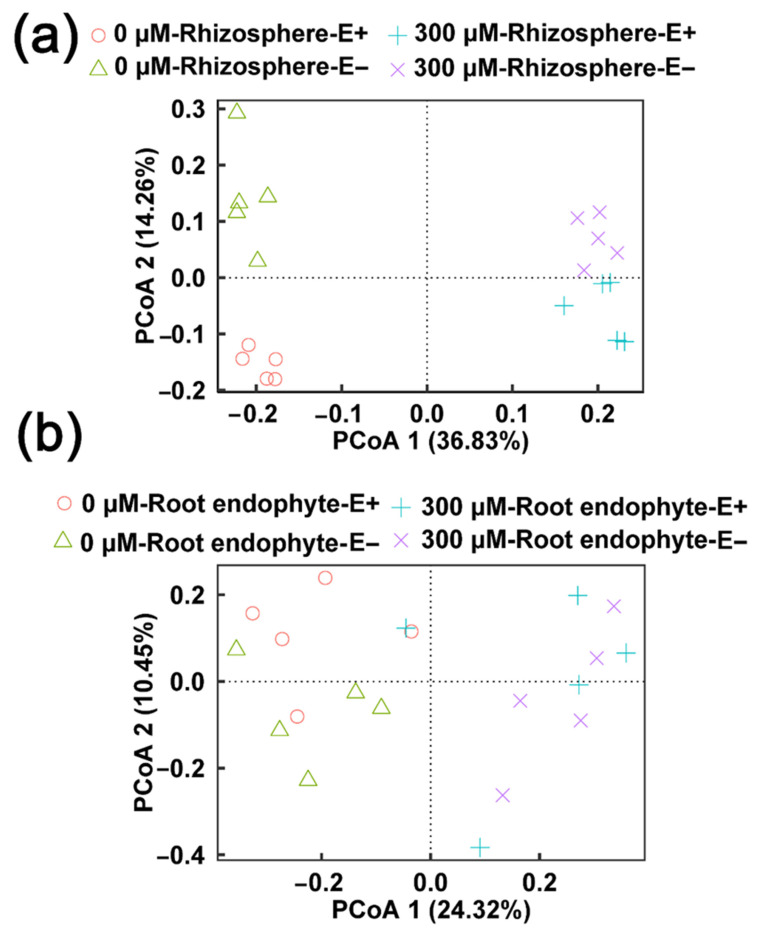
Principal coordinate analyses (PCoA) of bacterial community in rhizosphere (**a**) and root endosphere (**b**) of E+ and E− plants based on Bray–Curtis under 0 and 300 μM CdCl_2_ concentration.

To explore how the bacterial community varied with CdCl_2_ concentrations, sampling site (rhizosphere, root) and *E. gansuensis*-infection analyses were carried out with a PCoA on the calculated Bray–Curtis distances. As shown in Figure 3a, the control groups were distributed on the left side of the PCoA plot, while the 300 μM CdCl_2_ treatment groups were concentrated on the right side of the PCoA plot. The first and second principal coordinate explained 36.83% and 14.26% of the total variation of the bacterial community of the rhizosphere in E+ and E− *A. inebrians* at different treatment conditions, respectively. The Adonis results showed obvious differences (R^2^ = 0.366, *p* = 0.001) between the control and CdCl_2_ treatment groups (Table 1). Samples of the *E. gansuensis*-infection (Adonis: R^2^ = 0.118, *p* = 0.002) cultivar were markedly separated from those of E− plant, suggesting that the rhizosphere bacterial community differed between E+ and E− plants, and the differences under Cd stress conditions were larger than those under control conditions. Similarly, the different CdCl_2_ concentrations (Adonis: R^2^ = 0.213, *p* = 0.001) were able to significantly separate the endophytic bacterial community of roots between the E+ and E− *A. inebrians* (Figure 3b), but *E. gansuensis*-infection (Adonis: R^2^ = 0.038, *p* = 0.595) did not markedly influence the endophytic bacterial community of roots in *A. inebrians* between 0 μM CdCl_2_ and 300 μM CdCl_2_ (Table 1). The result also supported the conclusion of the difference analysis (Figure 2d), and further indicated that *E. gansuensis*-induced recruitment of specific rhizosphere bacterial community appeared to be more sensitive to Cd stress than that of the endophytic bacterial community.

#### 2.2.2. Network Characteristics of Bacterial Community

The above-mentioned results mainly concentrated on the diversity and composition differences of microorganisms, but microbes coexist and interact with one another in the ecosystem through complicated network structures [31]. Network analysis could be used to explore the changes in co-occurrence patterns of microbial communities under various environmental conditions [32]. Here, the co-occurrence network analyses were employed to investigate the effects of CdCl_2_ and *E. gansuensis* on the interspecific ecological relationship.

**Figure 4:**
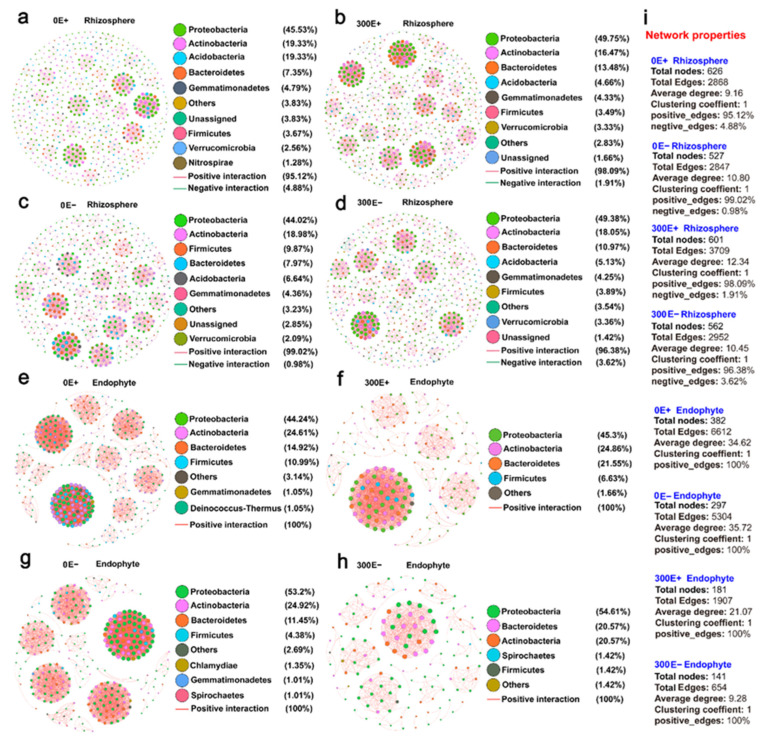
Co-occurrence networks of rhizosphere and endosphere bacterial community. (**a**) E+ rhizosphere at 0 μM CdCl_2_ treatment, (**b**) E+ rhizosphere at 300 μM CdCl_2_ treatment, (**c**) E− rhizosphere at 0 μM CdCl_2_ treatment, (**d**) E− rhizosphere at 300 μM CdCl_2_ treatment, (**e**) E+ root endosphere at 0 μM CdCl_2_ treatment, (**f**) E+ root endosphere at 300 μM CdCl_2_ treatment, (**g**) E− root endosphere at 0 μM CdCl_2_ treatment, (**h**) E− root endosphere at 300 μM CdCl_2_ treatment, (**i**) network properties.

The co-occurrence patterns of rhizosphere and endophytic bacteria were strongly affected by the CdCl_2_ treatments in E+ and E− plants (Figure 4). For the rhizosphere network (Figure 4a–d), the number of total nodes, total edges and an average degree in the E+ plant were all higher than those in the E− plant (Figure 4i), indicating a much greater complexity and connectedness in E+ *A. inebrians* when challenged by CdCl_2_. A recent study about the inoculation of the plant-growth-promoting rhizobacteria (PGPR) NSX2 in *Sedum plumbizincicola* under the Cd-contaminated soil also reached a similar conclusion [33]. A more complicated network means that microbial interactivities are more active and frequent, which would contribute to the phytoremediation of HMs in soils [33]. In addition, the proportion of positive correlations in E+ *A. inebrians* increased from 95.1% in the control condition to 98.1% in CdCl_2_ treatment, while those in E− plant decreased from 99.0% to 96.4% (Figure 4i). The positive correlations between species suggest that the ecological niches are the same or there is a close symbiotic relationship [34]. Yi et al. [28] similarly reported that bacterial community can enhance intraspecific cooperation under benzo[a]pyrene (BaP) stress. Therefore, the above results indicated that the *E. gansuensis* infection strengthened the cooperation between *A. inebrians* rhizosphere bacterial species to adapt to the Cd-contaminated environment.

In the endophytic network (Figure 4e–h), CdCl_2_ treatment decreased the numbers of total nodes, total edges and average degree, especially in E− plant, indicating that Cd stress decreased the complexity of the endophyte bacterial network structure. That is, when the external environment changed, the simpler network would faster transmit the environmental interference to the whole network, resulting in the instability of the network structure [35]. Consistent with previous studies [36], this result showed that Cd stress reduced the capacity of the endophytic network to buffer environmental disturbance. On the other hand, the result illustrated, from one perspective, that the endosphere environment was less directly disturbed than the rhizosphere environment under Cd stress. Similar findings were reported by Liu et al. [32], where the network density, total nodes and links among microbial communities declined under favorable soil environment. Moreover, the relatively low level of endophyte bacterial diversity compared to that in the rhizosphere was also responsible for the simpler network (Figure 2a).

#### 2.2.3. Response Characteristics of Bacterial Community Function

**Figure 5:**
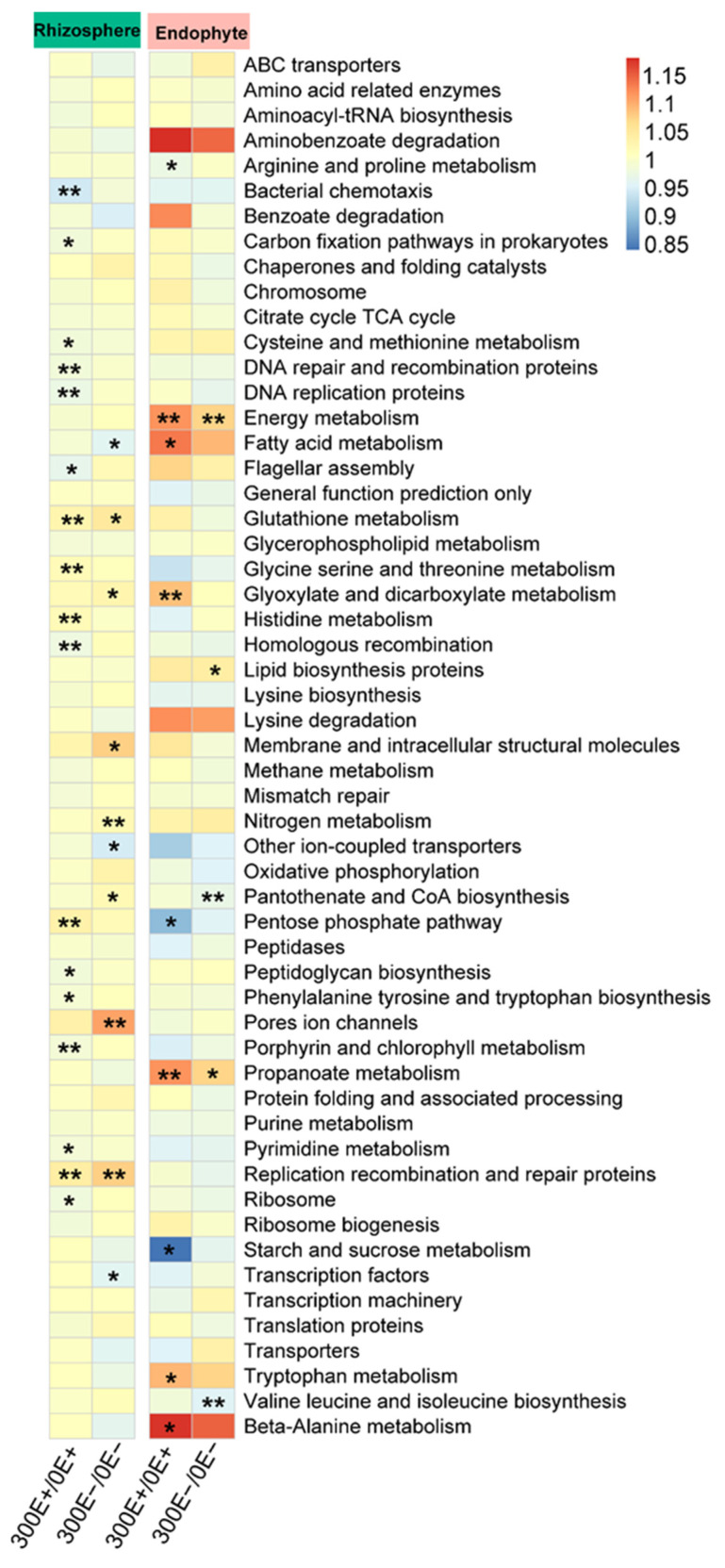
Heatmap clustering analysis of the predicted KEGG orthologs (KEGG level 3) in rhizosphere and endosphere bacteria across all samples. Asterisks indicated significant difference between 0 and 300 μM CdCl_2_ conditions in E+ and E− plants (* *p* < 0.05, ** *p* < 0.01).

Microbes with a variety of functions drive the bio-geochemical cycles of soil [37]. To further investigate the response of bacterial functionality to Cd stress, we performed the PICRUSt analysis. The dominant functions of level 1 were metabolism, genetic information processing and environmental information processing, which accounted for more than 70.0% both in E+ and E− plants (Table S1). A heat map was generated using the relative abundance of genes predicted in level 2 functional metabolic pathways (Figure S1). The results showed that samples in the endosphere and rhizosphere exhibited an opposite trend in level 2, suggesting that their functions were different. Similar results were also observed in a previous study [38], likely due to the different composition of microbes in the endosphere and rhizosphere. Analysis of the level 3 functional metabolic pathways indicated that most functions have changed significantly in rhizosphere bacteria after suffering CdCl_2_ stress (Figure 5). Although a quarter of the functions (25.0%, 17 out of 68 functions) and 14.7% (10 out of 68 functions) were obviously changed in E+ and E− plants, respectively, only two functions changed consistently between E+ and E− *A. inebrians*. Unexpectedly, in contrast to the rhizosphere, only a few functions showed significant shifts in the endosphere samples under Cd stress (Figure 5). Together, this result confirmed the Cd-induced shifts in bacterial functionality [35], which occurred primarily in the rhizosphere and differed between E+ and E− *A. inebrians*.

### 2.3. Root Exudate Profiles of E+ and E− A. inebrians under CdCl_2_ Stress

The rhizosphere is the part of soil closest to the plant roots where interactions between soil microbes and the plant play a key role in improving HMs tolerance of host [39]. Conversely, microorganisms can obtain energy from exudation secreted by plant root. As a result, plants actively affect the composition of the rhizosphere community by regulating root exudates [40]. To further explore the relationship between *E. gansuensis*-mediated changes in the bacterial community and *A. inebrians* root exudates, we initially analyzed the compositions of root exudates in the E+ and E− plants under 0 μM CdCl_2_ and 300 μM CdCl_2_ treatments.

**Figure 6:**
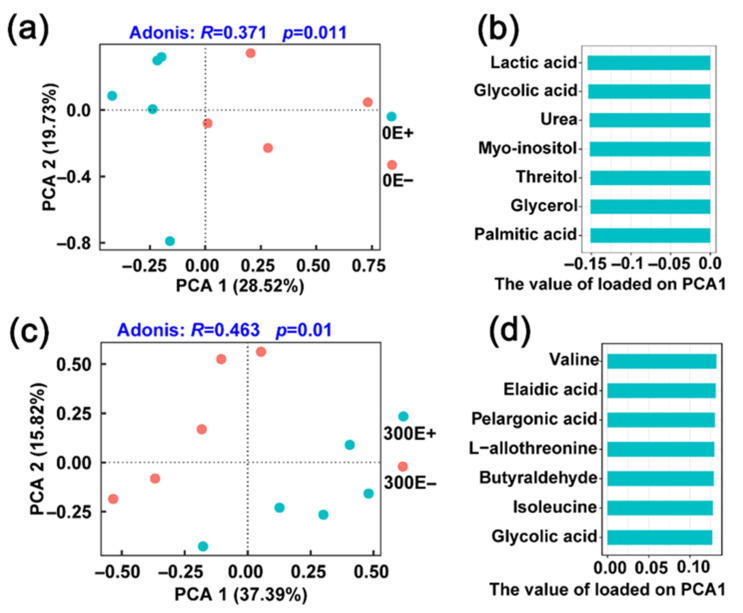
Principal component analysis (PCA) of root exudates profiles between E+ and E− plants under 0 μM CdCl_2_ condition (**a**) and 300 μM CdCl_2_ condition (**c**). (**b**) The top 7 (top 4% of total 139 compounds) compounds by the value of loaded on PCA1 of between E+ and E− plants under 0 μM CdCl_2_ condition (**d**) and 300 μM CdCl_2_ condition.

A total of 298 chromatographic peaks were found, and we identified 139 metabolites from all the samples. PCA of the profiles of the 139 compounds indicated that the root exudates of the E+ and E− plants were different whether grown under control or CdCl_2_ treatment conditions (Figure 6). For the control condition, the first axis of the PCA explained 28.5% of the variance. The top seven compounds (lactic acid, glycolic acid, urea, myo-inositol, threitol, glycerol, palmitic acid) (top 4% of total 139 compounds) from PCA1 were important to distinguish the exudate profiles of E+ and E− *A. inebrians* (Figure 6a,b). Similarly, the root exudates of E+ plant clearly separated from those of E− plant when challenged by Cd stress (Adonis: R = 0.463, *p* = 0.01), with the loading matrix of the PCA1 explained 37.4% of the variance (Figure 6c). The top seven metabolites, including valine, elaidic acid, pelargonic acid, L-Allothreonine, butyraldehyde, isoleucine and glycolic acid, from PCA1 were identified to distinguish the exudate profiles of E+ and E− plants (Figure 6d).

**Figure 7:**
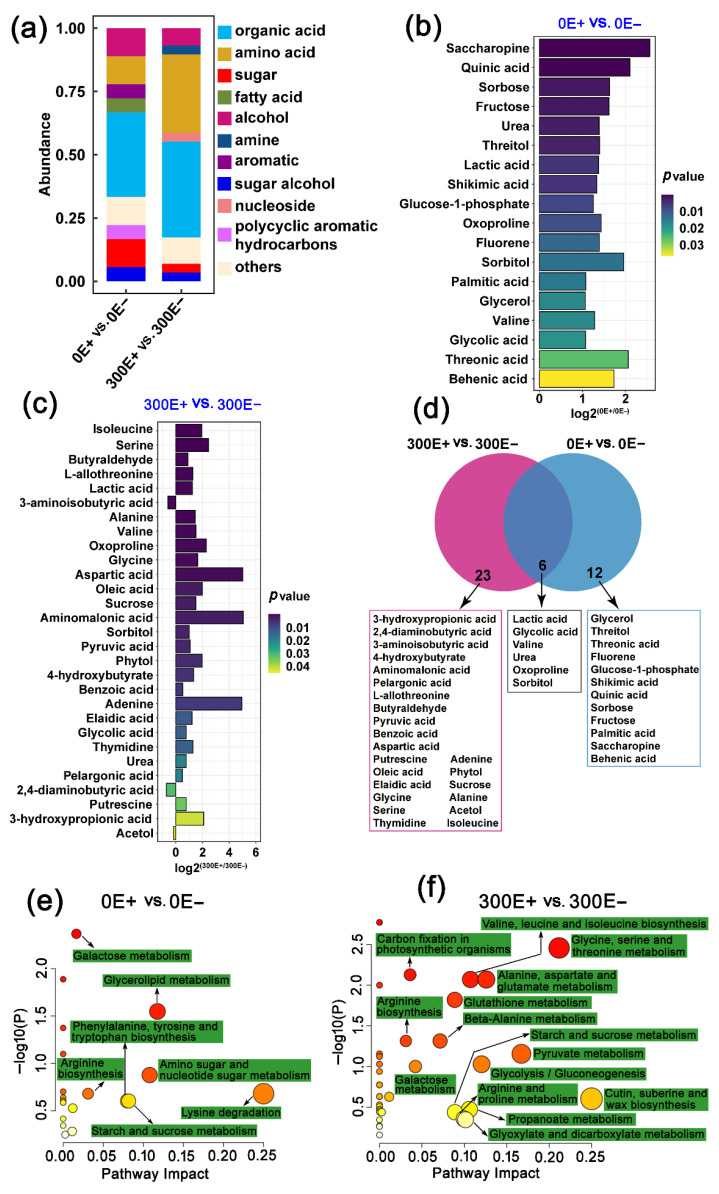
Effect of *E. gansuensis* infection on composition and functional feature of root exudates in *A. inebrians* under different CdCl_2_ conditions. (**a**) Relative abundance (%) of root exudates. (**b**) The differential root exudates between E+ and E− plants under 0 μM CdCl_2_ treatment (**c**) and 300 μM CdCl_2_ treatment. (**d**) Venn diagram showing the differential root exudates. (**e**) KEGG pathway enrichment analysis of the differential root exudates between E+ and E− plants under 0 μM CdCl_2_ treatment (**f**) and 300 μM CdCl_2_ treatment. The colors of circle in the ordinate represents the *p* value of enrichment analysis, with darker color indicating a more significant enrichment degree.

To accurately obtain the metabolite features of the root exudates, differential metabolites (DMs) (*p* < 0.05) were selected to further analysis. As shown in Figure 7, there were 18 and 29 compounds that were significantly different between the exudates of E+ and E− *A. inebrians* at 0 μM CdCl_2_ and 300 μM CdCl_2_, respectively. Specifically, the 18 DMs were divided into several categories with their chemical nature, namely, organic acids (6), amino acids (2), sugars (2), alcohol (2), fatty acid (1), polycyclic aromatic hydrocarbons (1), sugar alcohol (1), aromatic (1), others (2) (Figure 7a,b), and the 29 DMs included organic acids (11), amino acids (9), sugars (1), alcohol (2), amine (1), nucleoside (1), sugar alcohol (1), others (3) (Figure 7a,c). It was noteworthy that the relative contents of various organic acids (pyruvic acid, 3-hydroxypropionic acid, 4-hydroxybutyrate, benzoic acid, pelargonic acid, oleic acid, elaidic acid, aminomalonic acid) and amino acids (alanine, isoleucine, glycine, serine, L-allothreonine, aspartic acid) in the E+ plant were obviously higher than those in the E− plant under 300 μM CdCl_2_ treatment condition (Figure 7c). Previous studies have shown that the secretion of organic acids and amino acids enhanced the Cd tolerance of maize and rice cultivars by improving the nutrient uptake, plant growth and antioxidant in Cd-contaminated soil [41,42]. Therefore, the changes of root exudates might be the external detoxification and phytostabilization strategy of E+ *A. inebrians* to Cd contamination. On the other hand, many root exudates, such as sugars, amino acids, organic acids, could prevent the entry of Cd to the root cells or form chelate compounds with Cd at the soil–root interface to avoid Cd toxicity in plant tissues [43]. This supports our result that the E+ plant had less Cd accumulation than the E− plant (Figure 1b). Moreover, six metabolites (lactic acid, glycolic acid, valine, urea, oxoproline, sorbitol) were significantly upregulated by *E. gansuensis*, but not affected by CdCl_2_ stress (Figure 7d). In addition, further analysis of metabolic pathways indicated that multiple amino acid metabolism pathways, including glycine, serine and threonine metabolism, alanine, aspartate and glutamate metabolism, valine, leucine and isoleucine biosynthesis, beta-alanine metabolism, arginine and proline metabolism and glutathione metabolism were regulated by *E. gansuensis* under Cd stress (Figure 7f). The results indicated the variations of root exudates mediated by *E. gansuensis* that were actively involved in combating Cd toxicity and enhancing Cd tolerance in *A. inebrians*. Similar results were obtained in maize plants [44].

### 2.4. Relationship of Bacterial Community with Root Exudates under CdCl_2_ Stress

**Figure 8:**
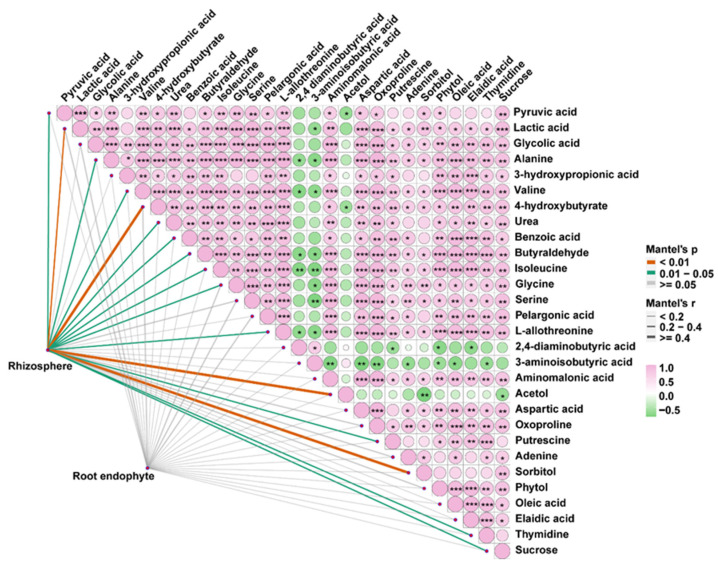
Relationship between differential root exudates and OTUs number of rhizosphere and root endosphere bacteria. Asterisks indicated statistically supported differences (* *p* < 0.05, ** *p* < 0.01, *** *p* < 0.001).

**Figure 9:**
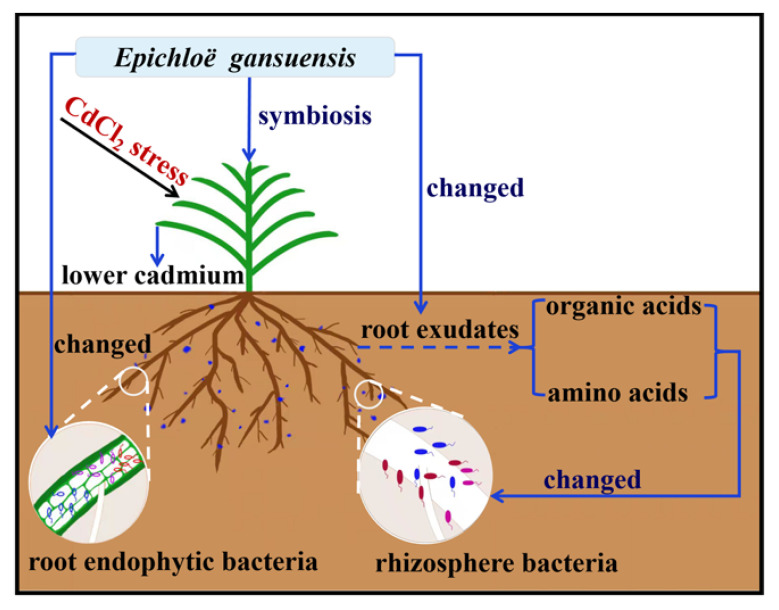
A working model of the *E. gansuensis* infection for improving Cd tolerance of *A. inebrians*.

The way in which *E. gansuensis* infection assists in recruiting beneficial microorganisms of the host is the key to reveal the mechanisms that improve the Cd resistance of symbionts. Therefore, Pearson’s correlation analyses of bacterial community and DMs under CdCl_2_ stress were investigated to explore their possible links. As shown in Figure 8, significant correlations were observed among more than half of the DMs (55.2%, 16 out of 29 DMs) including 4-hydroxybutyrate, sucrose, acetol, putrescine, glycine, pyruvic acid with OTUs in the rhizosphere bacterial community. Root exudates secreted by plants play big roles not only in changing metal bioavailability, but also attract various microbes by providing nutrient and energy sources [45]. Some of the sugars and organic acids with stimulated production in the CdCl_2_ treatment were reported to provide carbon sources for microbial communities, such as sucrose and pyruvic acid [46]. Putrescine, glycine, L-allothreonine and isoleucine could be served as nitrogen sources to satisfy the nitrogen demands of microbial communities [47]. Unlike the rhizosphere bacterial community; however, no significant correlation was found between root exudates and OTUs in the endosphere bacterial community (Figure 8), suggesting DMs had fewer effects on the endosphere bacterial community. It was probably because the rhizosphere is a narrow part of soil directly affected by root exudates [48], while the endosphere environment is relatively stable and is not affected by internal and external factors in the short term. Taken together, these results indicated the strategy of changing root exudates employed by E+ plant to recruit beneficial rhizosphere bacteria, rather than the endosphere bacteria, for alleviating Cd stress (Figure 9).

## 3. Materials and Methods

### 3.1. Plant Growth and Cd Treatment

*Epichloë gansuensis* infected (E+) and uninfected (E−) *Achnatherum inebrians* seeds were sterilized with 75% ethanol for 5 min and 1% NaClO for 10 min, and then rinsed 5 times with sterile water. Before plantation, 24 pots (10 cm in lower diameter ×18.5 cm in upper diameter ×19.5 cm in height) were filled with 2.4 Kg soil, collected from a fallow experimental field at the Yuzhong campus of Lanzhou University, which was passed through a 2 mm sieve. The E+ and E− *A. inebrians* seeds were sown in 12 pots, respectively, with six seeds per pot. The basic properties of the soil were as follows: pH: 8.40; total organic carbon: 3.50 g kg^−1^; total N: 1.17 mg g^−1^; soil ammonium N: 0.0116 mg g^−1^; soil nitrate N: 0.0016 mg g^−1^; total P: 0.624 mg g^−1^; available P, 0.003 mg g^−1^. One week after germination, 3 robust seedlings of similar size were retained in each pot. Each pot was provided 200 mL of distilled water once a week and a total of 2.4 L distilled water was added to each tray a week. After 20 days of growth, 6 E+ pots and 6 E− pots were treated with 200 mL 0 μM CdCl_2_ and 6 E+ pots and 6 E− pots were treated with 200 mL 300 μM CdCl_2_. Similarly, 2.4 L distilled water with 300 μM CdCl_2_ was applied to each tray receiving the CdCl_2_ in a week. The treatments received weekly applications of 300 μM CdCl_2_ or distilled water for 16 weeks. After 16 weeks of CdCl_2_ treatment, all E+ and E− plants continued to grow in the greenhouse (16 h light/8 h dark, humidity: 60%, temperature: 25 °C) for 8 months. The rhizosphere soil and roots of E+ and E− *A. inebrians* were then obtained, respectively, and put into ice before storing at −80 °C until DNA extraction.

### 3.2. Determination of the Dry Weight and Cd Content of Leaf and Root

By the end of the experiments, the leaf and root of E+ and E− plants were separated and dried at 80 °C to determine the dry weight. The Cd content of leaf and root in E+ and E− plants were determined by the atomic absorption spectroscopy method after digestion according to the method described by Qin et al. [49]. Briefly, *A. inebrians* samples (0.1 g) were put into a sealed polytetrafluoroethylene tube and an acid mixture (HNO_3_-H_2_O_2_ in a ratio of 4:1) was added to the automatic digester (Mars 6, CEM, USA) for digestion. After the digestion was completed, the solution cooled to room temperature was fixed to a 25 mL volumetric flask for determination the Cd concentration by an atomic absorption spectrometer (ZEEnit700P^®^, Analytikjena, Germany).

### 3.3. Acquisition of Rhizosphere Soil and Surface Disinfection of Roots

To obtain rhizosphere soil, the root samples of *A. inebrians* plants were collected from each E+ and E− pot. For every pot, individual root samples of three *A. inebrians* were mixed into a composite. Further, we followed the procedure of McPherson et al. [50] and Tang et al. [51] to get the rhizosphere soil, and to surface sterilize the roots. The rhizosphere soil and surface-sterilized roots were used for the analysis of the bacterial community of rhizosphere soil and the endophytic bacteria of roots in E+ plants and E− plants.

### 3.4. DNA Extraction, Amplicon Sequence and Bioinformatics Analysis

The cDNA libraries were sequenced with Illumina 2 × 250 bp platform, and the adaptor and primer sequences were removed from the sequences by using the cutadapt [52] plug-in of QIIME2 [53] software. In order to obtain high-quality sequencing data and improve the accuracy of subsequent bioinformatics analysis, the DADA2 plug-in of QIIME2 software was used to filter the data, denoise, merge and remove chimera [54]. The remaining clean reads were then processed into amplified sequence variant (ASV) by using the DADA2 plug-in of QIIME2 software.

### 3.5. Root Exudates Determination

The seeds of E+ and E− *A. inebrians* were surface sterilized, as mentioned above, and sown in a tray filled with sterilized vermiculite (150 °C for 3 h). After germination, E+ and E− *A. inebrians* trays were treated with 400 mL 1/2 Hoagland solution and 400 mL sterilized water every week. The E+ and E− plants trays were kept in a growth chamber (16 h light/8 h dark, humidity: 60%, temperature: 25 °C) for 45 days. Afterward, the plants were carefully taken out of the vermiculite, and the vermiculite was washed at the root with tap water. Next, E+ and E− seedlings were carefully transferred to 1/2 Hoagland solution in a 50 mL sterile centrifuge tube and allowed to grow for another day. Later, E+ and E− seedlings were treated with sterile 1/2 Hoagland solution with 0 and 300 μM CdCl_2_ for three days, and replaced the nutrient solution with different CdCl_2_ concentrations every day. To get root exudates, the roots of E+ and E− seedlings were washed with sterile water; E+ and E− seedlings were carefully transferred into conical bottles containing 20 mL of sterile water (one seedling per bottle) and allowed to grow for three more days. The conical bottles were shaken at 50 rpm for 2 h every day. Finally, 19 samples of root exudates were obtained and stored at −80 °C, and then dried in a lyophilizer (LNG-T98, Taicang Huamei biochemical instrument factory, China). Each cultivar was grown in five replicates (E− seedlings at 0μM CdCl_2_ were for four replicates), with one replicate including two seedlings. All the conical bottles were randomized in the greenhouse. For extraction, samples were transferred into a 50 mL tube for freeze-drying, a pre-cold extraction mixture in prorated (methanol) with internal standard (adonitol, 0.5 mg/mL stock) was added, vortexed for 30 s and ultrasonicated for 10 min (ice bath). After centrifugation at 12,000 rpm (RCF = 13,800 g, R = 8.6 cm) for 15 min at 4 °C, 400 μL of the supernatant was transferred to a new tube. In order to prepare the QC (Quality control) samples, 100 μL sample solution from each sample was collected and mixed. After evaporation in a vacuum concentrator, 30 μL of Methoxyamination hydrochloride (20 mg/mL in pyridine) was added, followed by incubation at 80 °C for 30 min, and then derivatized by 40 μL of BSTFA regent (1% TMCS, *v*/*v*) at 70 °C for 1.5 h. Later, samples were gradually allowed to cool to room temperature and added 5 μL of FAMEs (in chloroform) to the QC sample. All samples were then analyzed by gas chromatography coupled with a time-of-flight mass spectrometer (GC-TOF-MS). The GC-TOF-MS analysis and raw peak analysis were performed as reported by Hou et al. [55].

### 3.6. Statistical Analysis

The significant difference between E+ and E− plants at 0 and 300 μM CdCl_2_, respectively, for the dry weight of leaf and roots, and Cd content was performed by independent *T*-tests, and the significant differences were at *p* < 0.05. The Shannon and Chao1 indices of bacterial communities of rhizosphere and roots were carried out. The relative abundance of bacterial communities of rhizosphere and roots at phyla and genus level, principal coordinate analysis (PCoA), heatmap, co-network of bacterial communities of rhizosphere and roots at phyla level, the relationship between differential root exudates and OTUs number of rhizosphere and root endosphere bacteria were determined with R (4.1.3 Version). To estimate the potential function of bacteria, the PICRUST was used at Galaxy/Hutlab (https://huttenhower.sph.harvard.edu/galaxy/). For the analyses of root exudates, principal component analysis (PCA) was used with the R (4.1.3 Version). In addition, the differential root exudates were identified by using the Student’s *t*-test (*p* < 0.05), the analysis of metabolic pathways was performed via MetaboAnalyst5.0 (https://www.metaboanalyst.ca/ accessed on 6 March 2022).

## 4. Conclusions

The *E. gansuensis* infection improved the Cd tolerance of the host, which was mainly reflected in the increase in dry weight and the decrease in Cd content in plant tissues. The rare species were more strongly affected than the abundant species, especially in the rhizosphere bacterial community. The Cd-tolerant phyla of rhizosphere bacteria induced by *E. gansuensis* in this study were Nitrospirae, Chlamydiae and Deinococcus-Thermus, whose relative abundance increased. For the co-occurrence patterns, the *E. gansuensis*-infection enhanced the rhizosphere bacterial community interspecific cooperation to cope with Cd stress. Moreover, there were significant differences in root exudates between the E+ and E-plants when challenged by CdCl_2_ stress. On the one hand, the increase in amino acids and organic acids in root exudates induced by *E. gansuensis* contributed to promoting plant growth under Cd stress. On the other hand, the change of root exudates was conducive for the E+ plant to recruit the beneficial rhizosphere bacteria to alleviate Cd stress. The current findings might be essential to further improve the phytostabilization efficiency by using symbionts.

## Figures and Tables

**Table 1:** The statistical test of adonis to analyze the differences of bacterial community of rhizosphere and root endosphere in *E. gansuensis* infection and CdCl_2_ treatment.

Treatments	Rhizosphere		Root Endophyte	
	R^2^	*p*	R^2^	*p*
*E. gansuensis*	0.082	0.025	0.062	0.056
CdCl_2_	0.287	0.001	0.211	0.001
*E. gansuensis* × CdCl_2_	0.064	0.040	0.069	0.054

## Data Availability

The data presented in this study are available in the article and supplementary material.

## Supplementary Materials

The following supporting information can be downloaded at: https://www.mdpi.com/article/10.3390/ijms232113094/s1.
