# Supplementary material for: Increase in Cd Tolerance through Seed-Borne Endophytic Fungus Epichloë gansuensis Affected Root Exudates and Rhizosphere Bacterial Community of Achnatherum inebrians
Source: Int J Mol Sci. 2022 Oct 28;23(21):13094. doi: 10.3390/ijms232113094 (PMC9654189; doi:10.3390/ijms232113094)
Supplement: Supplementary file 1 [file ijms-23-13094-s001.zip › ijms-1970469-supplementary.pdf]

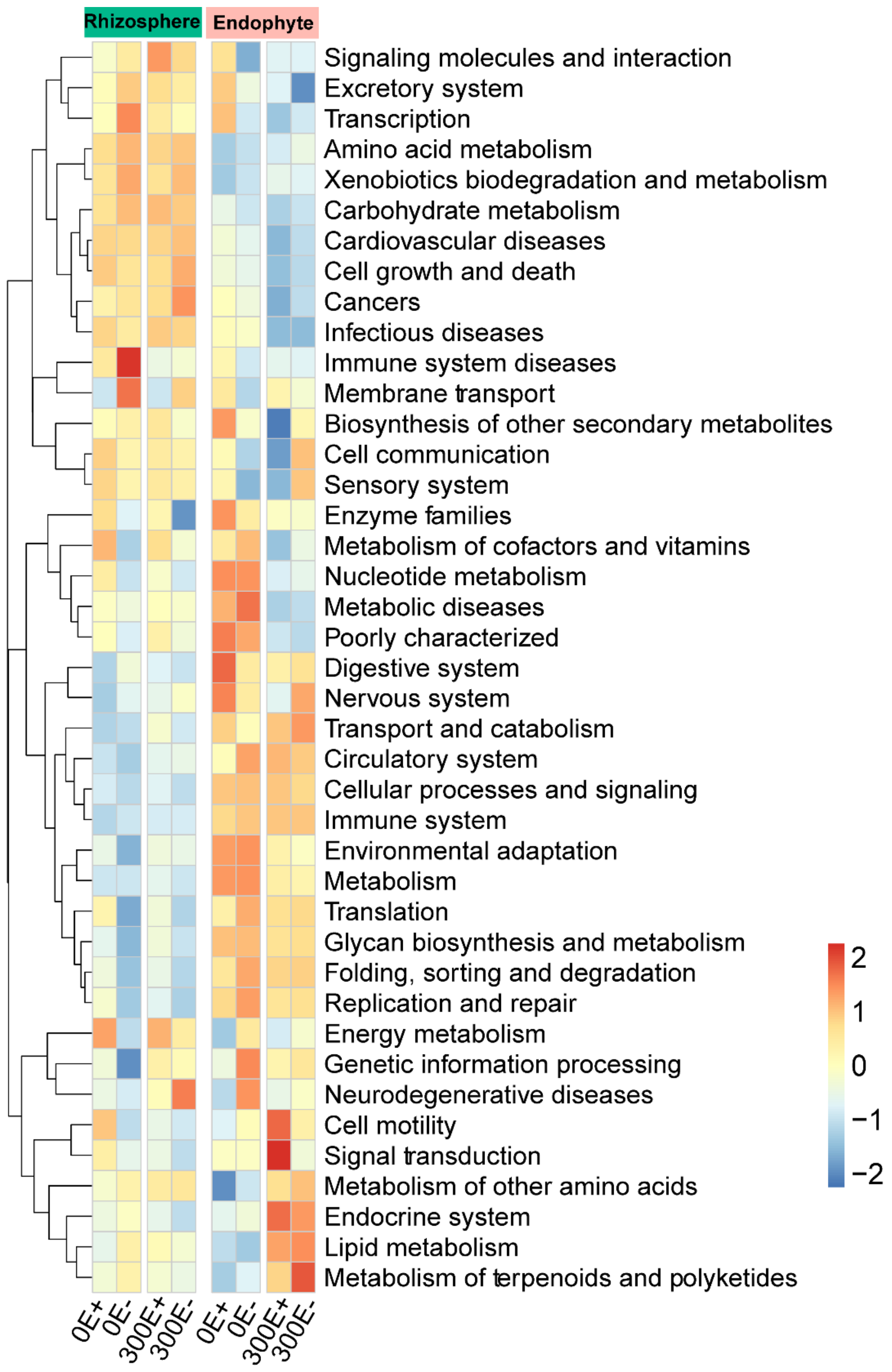

Figure S1. Heatmap clustering analysis of the predicted KEGG orthologs (KEGG level 2) in rhizosphere and endosphere bacteria across all samples.

Table S1. Relative abundance (%) of predicted KEGG Orthologs (KEGG level 1) in rhizosphere and endosphere bacteria across all samples.

Level 1	Rhizosphere				Root endophyte			
	0E+	0E-	300E+	300E-	0E+	0E-	300E+	300E-
Metabolism	0.508	0.507	0.510	0.508	0.496	0.498	0.497	0.501
Genetic Information Processing	0.154	0.149	0.152	0.149	0.157	0.160	0.157	0.158
Environmental Information Processing	0.139	0.149	0.139	0.146	0.144	0.139	0.144	0.142
Cellular Processes	0.043	0.041	0.042	0.042	0.042	0.042	0.043	0.042
Human Diseases	0.011	0.011	0.011	0.011	0.011	0.011	0.010	0.010
Organismal Systems	0.008	0.008	0.008	0.008	0.008	0.009	0.009	0.009
Unclassified	0.136	0.135	0.137	0.136	0.141	0.142	0.140	0.139
